# Welcome to the World of Zinc Signaling

**DOI:** 10.3390/ijms19030785

**Published:** 2018-03-09

**Authors:** Toshiyuki Fukada, Taiho Kambe

**Affiliations:** 1 Molecular and Cellular Physiology, Faculty of Pharmaceutical Sciences, Tokushima Bunri University, Tokushima 770-8514, Japan; 2 Division of Pathology, Department of Oral Diagnostic Sciences, School of Dentistry, Showa University, Tokyo 142-8555, Japan; 3 RIKEN Center for Integrative Medical Sciences, Yokohama, Kanagawa 230-0042, Japan; 4 Division of Integrated Life Science, Graduate School of Biostudies, Kyoto University, Kyoto 606-8502, Japan

Zinc, an essential trace element, plays indispensable roles in multiple cellular processes. It regulates a great number of protein functions, including transcription factors, enzymes, adapters, and growth factors, as a structural and/or catalytic factor. Recent studies have highlighted another function of zinc as an intra- and intercellular signaling mediator, which is now recognized as the “zinc signal”. Indeed, zinc regulates cellular signaling pathways, which enables the conversion of extracellular stimuli into intracellular signals, and controls various intracellular and extracellular events. Thus, zinc mediates communication between cells. The zinc signal is essential for physiology, and its dysregulation causes a variety of diseases such as diabetes, cancer, osteoarthritis, dermatitis, and dementia. This indicates that the “zinc signal” is an emerging topic that will assist in our understanding of the nature of physiology and pathophysiology.

This special issue, "Zinc Signaling in Physiology and Pathogenesis" has two main goals. The first is to update the current information available about the crucial role of zinc signaling in biological processes on both a molecular and a physiological level. This will assist in addressing the questions underlying this unique phenomenon and discerning its future direction through the publishing of review articles by experts, as well as original papers. The second aim is to feature the 5th Meeting of the International Society for Zinc Biology 2017 (ISZB-2017) in collaboration with Zinc-Net (COST Action TD1304), held in Cyprus. As Lowe and Moran reported, ISZB-2017 was held in conjunction with the final dissemination meeting of the Network for the Biology of Zinc (Zinc-Net) at the University of Central Lancashire’s Cyprus campus in June 2017, with over 160 participants, 17 scientific symposia, four plenary speakers, and two poster discussion sessions [1]. Much of the research presented at this meeting had never been presented or published before, and the most of the authors featured in this special issue presented their research at this meeting. This means that this issue contains the most up-to-date information on zinc signaling and related biology. Twelve review articles by such invited authors, which have been included in this issue, are mentioned below.

From a molecular and biochemical point of view, the first article by Maret provides an overview of the regulatory functions of zinc signaling through its interaction with Ca^2+^, redox, and phosphorylation signaling, thus enabling the transmission of information within cells and communication between cells [2]. The article by Kambe et al. summarizes the various zinc transporters, i.e., the family of zinc transporters (ZNTs) and Zrt- and Irt-like proteins (ZlPs), and discusses the roles of these transporters in the early secretory pathway [3]. Further, Takagishi et al. review an update of zinc transporters and zinc signaling. They focus on the recent progress in determining the roles of SLC39A/ZIP family members in vivo [4]. Sunuwar et al. focuses on ZnR/GPR39, a G-protein coupled receptor that senses changes in the concentration of extracellular zinc, reviewing its physiological role in skin and the colon, as well as its implication in cancer [5]. In addition, Subramanian Vignesh and Deepe provide an overview of the current understanding of a family of metal-binding proteins, metallothioneins (MTs), especially focusing on their role in immunity [6].

From a viewpoint of physiology and medicine, Takeda and Tamano describe the impact of synaptic zinc signaling on cognition and its decline [7]. Portbury and Adlard highlight the role of zinc signaling in the central nervous system, and its potential implications in brain diseases such as cognitive decline, depression, and Alzheimer’s disease [8]. Fukunaka and Fujitani emphasize the contribution of zinc homeostasis on the pathophysiology of metabolic diseases, by focusing on the zinc transporters ZnT8 and ZIP13 [9]. Maywald et al. review the critical role of zinc homeostasis in the immune system. In addition, they describe the molecular mechanisms and targets that are affected by altered zinc homeostasis and illustrate several types of zinc signaling that are involved in the immune system [10]. Pyle et al. describe the possible molecular relationship between tuberculosis and zinc homeostasis. They also review the protective role of the zinc transporter ZIP8 in macrophages in *Mycobacterium tuberculosis* infection [11]. Turan and Tuncay review the current understanding of the physiological role of zinc signaling on heart functions and related diseases [12]. Cherasse and Urade suggest the potent connection between zinc status and sleep, and investigate its molecular mechanisms [13].

In addition to the reviews mentioned above, five research articles are included in this special issue. Hence, we must emphasize that this special issue will provide new insights into the role of zinc signaling, mediated by zinc transporters and zinc-binding proteins, in health and disease from a molecular to a physiological level. We hope that this will present our readers with novel opportunities to raise new ideas and connections to resolve persisting questions in the future. Finally, we would like to express our heartfelt gratitude to all of the authors and referees for their tremendous efforts in supporting this special issue. Without their valuable assistance, we would not have had even a glance of this timely and successfully publication with its useful updates on zinc signaling biology.
